# Nutritional Efficiency of *Coffea canephora*: The Role of Genetic Variability and Nutrient Accumulation

**DOI:** 10.3390/plants14101509

**Published:** 2025-05-17

**Authors:** Cleidson Alves da Silva, Jéssica Rodrigues Dalazen, Weverton Pereira Rodrigues, Rodrigo Barros Rocha, Fábio Luiz Partelli

**Affiliations:** 1 Campo Experimental de Patrocínio, Empresa de Pesquisa Agropecuária de Minas Gerais (EPAMIG), Patrocínio 38740-000, MG, Brazil; 2 Centro Universitário Norte do Espírito Santo, Universidade Federal do Espírito Santo, São Mateus 29932-540, ES, Brazil; jessica_dalazen@hotmail.com; 3 Centro de Ciências Agrarias, Universidade Estadual da Região Tocantina do Maranhão, Imperatriz 65900-001, MA, Brazil; weverton.rodrigues@uemasul.edu.br; 4 Brazilian Agricultural Research Corporation (EMBRAPA Coffee), Brasília 70770-901, DF, Brazil; rodrigo.rocha@embrapa.br; 5 Instituto Capixaba de Pesquisa, Assistência Técnica e Extensão Rural (INCAPER), Vitória 29052-010, ES, Brazil

**Keywords:** *Coffea canephora*, genotypes, nutrient accumulation, genetic divergence

## Abstract

The genetic variability of *Coffea canephora* is essential for the identification of genotypes with enhanced nutritional traits. This study aimed to characterize *C. canephora* genotypes based on nutrient accumulation in fruits, evaluated over two consecutive harvests. The experiment followed a randomized block design with four replications, comprising 42 genotypes. To assess nutrient accumulation, fruit samples were collected from each genotype and oven-dried. In a plant tissue analysis laboratory, the concentrations of N, P, K, Ca, Mg, S, Fe, Mn, Cu, Zn, and B were determined. Nutrient accumulation in the fruits was calculated as dry mass × nutrient concentration, and the data were converted to kg or g of nutrients accumulated per ton of coffee beans at 12% moisture content. The results revealed significant variability among genotypes in nutrient accumulation, with the general accumulation order being N > K > Ca > Mg > S > P > Mn > Fe > B > Cu > Zn. Multivariate analysis identified seven groups, with Verdim R, Clementino, and Pirata forming distinct clusters due to their unique characteristics. Clementino exhibited the highest nutrient accumulation, while LB1 had the lowest. The study demonstrated high heritability for all traits, indicating strong genetic control, along with significant positive correlations among nutrients. These findings highlight the potential of selecting nutrient-efficient genotypes to enhance the sustainability of coffee cultivation. The nutritional data obtained can support the development of more nutritionally efficient cultivars, ensuring long-term sustainability in coffee production.

## 1. Introduction

Coffee is one of the most widely consumed beverages worldwide. More than 130 coffee species have been identified [1]. However, the coffee used globally, either as a beverage or in processed products, is predominantly derived from two species: *Coffea arabica* and *C. canephora*. Owing to their commercial significance, the majority of research on coffee cultivation is concentrated on these two species.

One of the main distinctions between these two species lies in their reproductive strategies. While *C. arabica* is a self-pollinating species, *C. canephora* exhibits self-incompatibility, resulting in greater genetic variability within this species [2,3]. This high level of genetic diversity has been explored in numerous studies. In Brazil, research on the genetic variability of *C. canephora* is well documented, encompassing morpho-anatomical traits [4,5,6], floral characteristics [7,8], and molecular markers [9]. Previous studies on *C. canephora* have highlighted genotype-specific nutrient dynamics. Dubberstein et al. [10] linked increased nutrient accumulation to fruit growth, while Silva et al. [11] revealed genetic variability in leaf nutrient concentrations. Rodrigues et al. [12] and Schmidt et al. [13] further demonstrated genetic diversity in fruit nutrient accumulation. Global efforts to characterize *C. canephora*’s genetic diversity reveal its broad adaptive potential: wild populations with unique traits in the Democratic Republic of Congo [14,15], climate-resilient genotypes in Uganda [16,17], agronomic trait assessments in Ghana [18], SNP-based breeding strategies in India [19], and sensory profiling in Ecuador [20]. These efforts highlight the global genetic potential of the species.

Coffee cultivation has a high productive potential and, consequently, significant nutritional requirements. The low natural fertility of certain soils can be compensated for by the application of mineral nutrients, allowing for increased productivity. However, this practice also raises production costs and may compromise agricultural sustainability. Therefore, the study of plant mineral nutrition is essential for accurately assessing the nutritional needs of both soil and plants throughout the production cycle. This approach helps prevent improper fertilizer use, mitigates productivity constraints, and promotes sustainable agricultural practices [21].

During coffee fruit harvesting, nutrient export from the environment occurs—a natural and unavoidable process in agriculture. Understanding the magnitude of this process is essential for implementing appropriate management practices that ensure the long-term sustainability of coffee cultivation. By identifying the crop’s nutritional requirements and considering soil characteristics, effective strategies can be developed to maintain soil fertility and sustain high productivity over time. Research focused on coffee plants with reduced energy and nutrient demands has driven efforts to identify mechanisms that improve nutritional efficiency [21]. Plants with enhanced capacity for nutrient absorption, translocation, and utilization are particularly important for cultivation in soils with low natural fertility [22].

The selection of new clones and the characterization of those already under commercial cultivation are central objectives of coffee breeding programs [23]. New cultivars or accessions are essential components of any crop improvement effort, as they serve as potential parent plants for the development of new breeding populations [16]. Therefore, the objective of this study was to characterize *C. canephora* genotypes based on nutrient export through the fruits. We hypothesize that *C. canephora* genotypes exhibit significant genetic variability in nutrient export efficiency, and that this variability can be harnessed to select genotypes with lower nutrient export, contributing to more sustainable cultivation practices and reduced dependence on external fertilizer inputs.

## 2. Materials and Methods

### 2.1. Experimental Area

The experiment was conducted over two crop seasons, 2016/2017 and 2017/2018, on a rural property in the municipality of Nova Venécia, in the northern region of Espírito Santo State, Brazil. The coffee plantation was established in 2014 under full-sun conditions and irrigated using a drip irrigation system, with a spacing of 1 m between plants and 3 m between rows, totaling 3333 plants per hectare. The area is located at 18°39′43″ S and 40°25′52″ W, at an average altitude of approximately 200 m, with an annual average temperature of 23 °C.

According to Köppen’s classification, the region’s climate is classified as Aw (tropical with a dry season) [24]. The soil in the area is classified as a dystrophic Red-Yellow Latosol with a clayey texture and undulating relief [25]. The chemical and physical attributes of the soil were determined through laboratory analyses at depths of 0–10, 10–20, 20–30, 30–40, 40–50, and 50–60 cm, as presented in Table 1.

The experiment was conducted in a randomized block design with four replications over two consecutive harvests. The treatments comprised 42 clonal genotypes of *C. canephora* (propagated vegetatively) (Table 2). Each experimental plot consisted of seven plants. Crop management in the experimental area followed technical recommendations for *C. canephora* cultivation, including weed control with brush cutters and herbicides, phytosanitary management, soil acidity correction through liming, and fertilizer application [26]. The coffee plants received 500, 100, and 400 kg ha^−1^ year^−1^ of N, P_2_O_5_, and K_2_O, respectively, applied according to plant requirements and phenological stages. Soil micronutrient levels were adjusted with annual applications of 2 kg ha^−1^ of Zn, 1.0 kg ha^−1^ of B, 2.0 kg ha^−1^ of Cu, and 10 kg ha^−1^ of Mn.

### 2.2. Fruits Samples and Nutrient Accumulation

During the coffee harvest period, fruit samples were collected from each genotype at the cherry ripening stage. The genotypes were classified according to their ripening patterns into four categories: early (180–210 days after flowering, DAF), medium (211–240 DAF), late (241–270 DAF), or super late (extending beyond 270 DAF). The same trees were sampled in both crop seasons (2016/2017 and 2017/2018) to ensure data consistency. The samples were then sent to the Coffee Research Laboratory at the Federal University of Espírito Santo, São Mateus campus, for processing.

The sample weights were measured using a precision balance and then placed in a forced-air oven at 50 °C, where they were dried until reaching a constant weight, approximately 170 h. After drying, the dry matter content of the fruits was determined using a precision balance (0.001 g), and the values were adjusted to 12% moisture (commercial standard).

The samples were analyzed to determine the nutrient concentration in the fruits. The analyzed nutrient concentrations correspond to the whole coffee fruit (pulp, parchment, and beans), as commonly exported during harvest. In a plant tissue analysis laboratory, nutrient concentrations were determined using validated protocols adapted from Silva et al. [29]. Nitrogen (N) was quantified via sulfuric acid digestion followed by titrimetric analysis. Phosphorus (P), potassium (K), calcium (Ca), magnesium (Mg), sulfur (S), iron (Fe), manganese (Mn), copper (Cu), and zinc (Zn) were extracted by nitro-perchloric acid digestion (HNO_3_:HClO_4_, 3:1 *v*/*v*) and quantified using inductively coupled plasma optical emission spectrometry (ICP-OES; Spectro Arcos, Kleve, Germany). Boron (B) was analyzed by dry ashing (550 °C for 4 h), followed by dissolution in 0.1 mol L^−1^ HCl and colorimetric detection with azomethine-H using a UV-1800 spectrophotometer (Shimadzu, Kyoto, Japan). Nutrient accumulation in the fruits was calculated based on the dry mass × concentration of each nutrient.

### 2.3. Statistical Analyses

The data on nutrient accumulation in the fruits, averaged over the two evaluated harvests, were subjected to analysis of variance using the F test (*p* < 0.01 and *p* ≤ 0.05). The ANOVA model considered the effects of genotypes (fixed) and blocks (random), with the dataset representing the biennial average of nutrient accumulation. The Scott-Knott algorithm (*p* ≤ 0.05) was applied to group the means of nutrient accumulation among genotypes.

Genetic analysis focused on nutrient accumulation traits to characterize each genotype. These traits served as a basis for estimating genetic parameters and performing multivariate analyses. Nutrient accumulation values were standardized per ton of processed grain (12% moisture) to ensure comparability among genotypes. The experimental coefficients of variation (CVe), the genetic coefficient of variation (CVg), and the genotypic coefficient of determination (H^2^) were estimated according to the following formulas:

$$CVe=100\times \frac{\sqrt{QMR}}{M}$$

$$CVg=100\times \frac{\sqrt{\hat{\Phi }g}}{M}$$

$$H^{2}=100\times \frac{MSG-MSR}{MSG}$$

where:

MSR: Mean squared residues;

M: population mean for the trait considered;

$\hat{\Phi }g$: quadratic component estimator associated with genotypic variation;

MSG: mean square of genotype.

For the genetic diversity analysis, Euclidean distance was used as a measure of dissimilarity, and genotype clustering was performed using the hierarchical Unweighted Pair Group Method using Arithmetic Averages (UPGMA). The number of groups was determined by establishing a cutoff point based on Mojena’s [30] methodology, which considers the relative size of fusion levels in the dendrogram. The contribution of the evaluated traits to genetic diversity was determined following the methodology proposed by Singh [31].

To assess the correlation between nutrient accumulation in the fruits, Spearman’s non-parametric correlation analysis was performed. A Principal Component Analysis (PCA) was performed to explore the multivariate variability in nutrient accumulation among genotypes. The analysis included the average values of nutrient accumulation (N, P, K, Ca, Mg, S, Fe, Mn, Cu, Zn, and B) in the fruits over two consecutive harvests. Data standardization was applied to ensure equal contribution of all variables. The loadings (variable contributions) and scores (genotype positions) were interpreted to assess the structure of genetic diversity. The results were visualized through a biplot combining components PC1 and PC2. All statistical analyses were conducted using the Genes software [32].

## 3. Results

### 3.1. Nutrient Accumulation

The results indicated significant variability among genotypes regarding nutrient accumulation in the fruits. A statistically significant difference was observed among genotypes at a 1% probability level using the F test for all evaluated nutrients (Table 3). Based on the overall mean of the genotypes, the following nutrient accumulation order was obtained: N > K > Ca > Mg > S > P > Mn > Fe > B > Cu > Zn.

The assessment of nutrient accumulation in the fruits over the two-year period revealed the formation of distinct groups: 5 for K; 6 for Ca; 7 for N and B; 9 for Mg, S, and Zn; 10 for P, Cu, and Mn; and 13 for Fe (Table 3). Among the identified groups, some genotypes stood out over the two-year average, exhibiting either higher or lower accumulation levels for multiple nutrients. The Clementino genotype was noteworthy for being among those with the highest accumulation of 9 out of the 11 evaluated nutrients (N, P, K, Ca, Mg, S, Fe, Cu, and B). Conversely, the LB1 genotype stood out for being among the groups with the lowest accumulation means for eight nutrients (N, P, K, Mg, S, Fe, Zn, and B).

### 3.2. Genetic Variability

For all evaluated nutrients, the coefficient of experimental variation (CVe) remained below 6%, ranging from 1.86% for P accumulation to 5.69% for Ca accumulation. The coefficient of genetic variation (CVg) varied from 6.75% for N accumulation to 30.66% for Mn accumulation. Heritability values exceeded 94% for all evaluated nutrients (Table 4).

**Figure 1:**
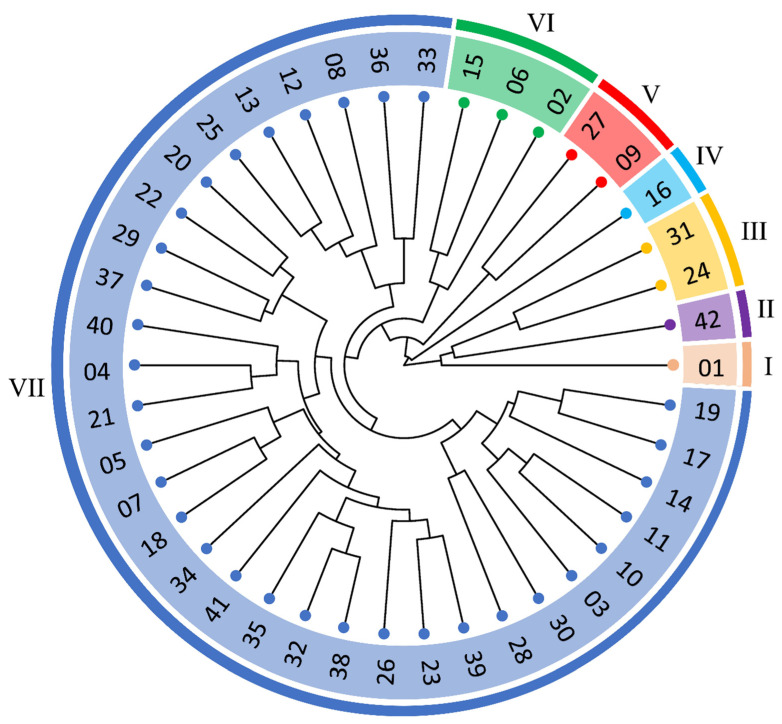
Dendrogram representing the dissimilarity among 42 *Coffea canephora* genotypes obtained using the UPGMA method with Euclidean distance, based on nutrient accumulation in the fruits of the genotypes during the 2017–2018 biennium for the production of one ton of processed coffee beans at 12% moisture. Roman numerals represent the number of groups formed. Cophenetic correlation: 0.68.

The clustering of genotypes using the UPGMA hierarchical method with Euclidean distance facilitated the formation of a dendrogram based on nutrient accumulation. By establishing a maximum limit of 71.39%, determined as the cutoff point using Mojena’s criterion, seven distinct groups were identified (Figure 1). Among these, the largest group comprised 32 genotypes, while three groups contained only a single genotype each. The genotypes Verdim R, Clementino, and Pirata were the most dissimilar, with each forming its own individual group.

The relative contribution of the variables to genetic diversity indicated that Mn and Fe accumulation were the most significant nutrients for predicting genetic diversity (Table 5). The combined accumulation of these two micronutrients accounted for 97.36% of the relative contribution to the prediction of the genetic diversity.

### 3.3. Correlation Among Nutrient Accumulations in the Fruit

**Figure 2:**
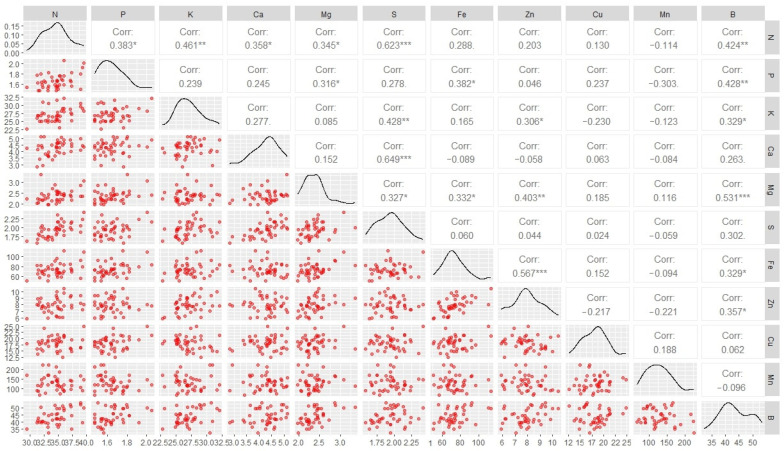
Correlations among the accumulations of N, P, K, Ca, Mg, S, Fe, Zn, Cu, Mn, and B in the fruits of *Coffea canephora* genotypes. *, **, and *** indicate significance levels of *p* < 0.05, *p* < 0.01, and *p* < 0.001, respectively.

The accumulation of all nutrients was correlated with one another, resulting in 55 correlations (Figure 2). Among these, 20 positive significant correlations were identified between nutrient accumulations in the fruits. Six significant correlations were found for N, Mg, and B; four for P, K, S, Fe, and Zn; two for Ca; and none for Mn and Cu.

### 3.4. Multivariate Principal Component Analysis (PCA)

**Figure 3:**
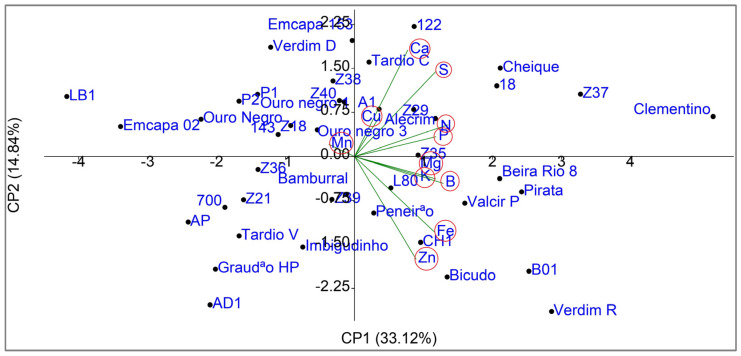
Biplot of Principal Component Analysis (PCA) representing the distribution of 42 *Coffea canephora* genotypes and the vectors of nutrient accumulation in the fruits (N, P, K, Ca, Mg, S, Fe, Mn, Cu, Zn, B), evaluated as the average of two consecutive harvests.

To explore the multivariate variability in nutrient accumulation, a Principal Component Analysis (PCA) was performed. The first two components (PC1 and PC2) explained 33.12% and 14.84% of the total variance, respectively, totaling 47.96% of the cumulative variability (Figure 3). PC1 was primarily associated with the accumulation of N, P, Mg, S, Fe, and B, while PC2 highlighted the positive contribution of Ca and S and the negative contribution of Zn. The distribution of the genotypes in the two-dimensional space (PC1 vs. PC2) revealed patterns consistent with the UPGMA analysis, with emphasis on the genotypes Clementino, LB1, and Verdim R, which occupied extreme positions in the biplot.

## 4. Discussion

The study identified genotypic variability in *C. canephora* for nutrient accumulation in fruits. Nutrient export resulting from fruit harvesting highlights the importance of this variability within the production system. The greater the nutrient export by the crop, the higher the requirement for nutrient replenishment through fertilization. Therefore, genotypes that exhibit lower nutrient export while maintaining above-average productivity are considered promising candidates for cultivation and for use in breeding programs.

It was found that N and K were the nutrients with the highest demand for the fruits, with average accumulations of 34 and 27 kg per ton of produced grains, respectively, across the evaluated genotypes and harvests. This demand for these nutrients by the fruits highlights the need for increased attention to replenishment through crop fertilization. Other authors have also noted that N and K are among the nutrients with the highest demand by the fruits [12,13]. The nutrient N is a component of many plant cellular structures, amino acids, and nucleic acids, while K is associated with the enzymatic activation of various metabolic processes, such as protein and carbohydrate synthesis, and is also involved in the transport of sugars from the source to the sink [33]. For coffee, the appropriate ratio of N to K influences beverage quality, being related to caffeine content, increased grain yield, total phenols, total and reducing sugars, and total acidity [34].

Based on the study results, it was possible to identify genotypes with the highest and lowest nutrient export per ton of grains produced. In this context, the genotypes Clementino and LB1 represent the extremes, exhibiting the highest and lowest nutrient accumulation, respectively. Comparatively, the genotype Clementino exports at least 30% more nitrogen (N) and phosphorus (P), and at least 40% more magnesium (Mg), sulfur (S), and boron (B) per ton of grains than the genotype LB1. These findings suggest that nutrient replenishment requirements in the field should be higher for the genotype Clementino.

The observation that the same genotype exhibited both the highest and lowest accumulation values for different nutrients, when compared to other genotypes, suggests that the nutritional dynamics of these genotypes influence multiple nutrients simultaneously. This indicates the presence of shared mechanisms governing nutrient absorption and export to the fruits. For example, the genotype LB1 demonstrated the lowest accumulation values for several nutrients, whereas the genotype Clementino exhibited the highest values. Furthermore, the correlations established among nutrients confirmed this pattern: positive associations indicate that genotypes with higher accumulation of one nutrient also tend to accumulate higher levels of other nutrients. Akpertey et al. [16] emphasize that the correlation between traits has received significant attention from plant breeders, primarily due to its importance in shortening breeding cycles. This approach enables optimization of selection gains for key traits in a shorter time frame while also reducing costs associated with collecting redundant data.

The analysis of nutrient accumulation correlations revealed a complex network of significant positive relationships, particularly for nitrogen (N), magnesium (Mg), and boron (B), which were each significantly correlated with six other nutrients. This pattern indicates that the physiological mechanisms regulating nutrient uptake, transport, and allocation are closely interconnected, potentially governed by shared genetic pathways or co-regulated transporters. Moreover, the absence of significant correlations for manganese (Mn) and copper (Cu) suggests distinct, independent regulatory mechanisms for these micronutrients. The strong genetic control observed for nutrient accumulation traits, reflected in high heritability estimates (exceeding 94%), confirms that these traits are predominantly influenced by genetic factors rather than environmental variability. Notably, iron (Fe) and manganese (Mn) demonstrated the highest contributions to genetic diversity, underscoring their potential role as indicators in breeding programs. The divergence of specific genotypes, such as Clementino and Verdim R, from the general clusters indicates genotype-specific nutrient absorption efficiencies and suggests opportunities for targeted selection of cultivars with optimized nutrient-use efficiency. These findings collectively support the development of breeding strategies aimed at enhancing nutrient-use efficiency while maintaining yield, thereby contributing to sustainable coffee production systems.

The variability observed in this study may be linked to differences in nutrient absorption processes, the compartmentalization performed by the roots and other plant organs, as well as the mobility of nutrients through the xylem and phloem vessels [35]. Furthermore, more recent studies have highlighted significant variability in the root characteristics of *C. canephora* genotypes [36,37], which can considerably influence the dynamics of nutrient absorption by the plants.

Another relevant aspect explaining the differences among genotypes in nutrient accumulation in the fruits is the variation in biomass accumulation rates in the fruits of *C. canephora* genotypes [13,38]. Genotypes with higher biomass accumulation rates may exhibit a dilution effect on nutrients, while those with lower biomass accumulation may demonstrate a concentration effect. Genotypes with higher and lower fruit yields may exhibit dilution and concentration effects, respectively. Dilution and concentration effects have also been considered in other studies involving *C. canephora* genotypes [11].

The genetic parameters indicated that genotypic variance (CVg) prevailed over environmental variance (CVe) for all nutrient accumulations studied. The values obtained for genotypic determination coefficient (H^2^) in nutrient accumulation indicated high genetic control over the traits, meaning they can be passed on to their offspring and thus utilized in breeding programs [16]. As nutrient absorption, transport, and redistribution are genetically controlled, it is possible to improve and/or select cultivars for more efficient nutrient use [39,40]. Estimates of genetic parameters allow breeders to better understand the nature of the genetic action involved in the inheritance of traits, provide a clearer assessment of the expected progress with selection, and help define the best selection strategies to be employed in any breeding program [41].

The multivariate analysis considering the accumulation of all nutrients in the fruits revealed the divergence among genotypes, forming seven dissimilar groups using the UPGMA clustering method. Three genotypes stood out by individually forming each dissimilar group: Verdim R, Clementino, and Pirata. The results of group formation highlight the divergence of these genotypes compared to others. The genotype Verdim R has the highest Zn accumulation value among all genotypes, while the genotype Pirata exhibits the highest Mg accumulation. The genotype Clementino, along with Verdim R, has the highest values for Fe accumulation among all genotypes. The largest group formed by the clustering method consisted of genotypes that obtained intermediate values for most nutrient accumulations. Using the hierarchical UPGMA method, other studies also observed the formation of divergent groups among *C. canephora* genotypes for nutritional traits [11,12,13,42,43]. This species is allogamous and self-incompatible, and these characteristics naturally favor the uniqueness of each genotype. The more heterogeneous the study group, the more pronounced the dissimilarities among individuals will be [3]. This study relied on phenotypic data (nutrient accumulation) to assess genetic diversity. Future studies integrating genotyping by sequencing or parentage matrices may refine clustering patterns and elucidate pleiotropic effects.

According to the method presented in the study, the micronutrients Mn and Fe emerged as the primary determinants in the formation of divergent groups using the UPGMA method. These elements exhibited the greatest variability among the genotypes, as evidenced by the higher number of groups formed in the Scott–Knott test and the elevated CVg values. The greater the CVg, the more heterogeneous the evaluated genotypes are [44]. Additional studies on genetic diversity in nutritional traits of *C. canephora* support these findings, highlighting the significant contribution of certain micronutrients to genetic diversity in the analyzed group [12,13,45].

The PCA complemented the analyses by revealing multivariate patterns in genetic divergence. While Singh’s method highlighted Mn as the main individual contributor to variability (87.2%), the PCA demonstrated that nutrients such as N, P, and S act synergistically in genotype differentiation (PC1), underscoring the importance of complementary approaches to identify key markers and explore complex interactions. The extreme positions of Clementino and Verdim R in the biplot corroborate their segregation in the UPGMA analysis, reinforcing their contrasting potential for breeding programs.

The results obtained in this study corroborate our initial hypothesis that *C. canephora* genotypes exhibit significant genetic variability in nutrient export efficiency. The identification of genotypes with contrasting nutrient accumulation profiles, such as Clementino and LB1, confirms the existence of substantial genetic variation influencing nutrient absorption and translocation to the fruits. Furthermore, the observed positive correlations among nutrient accumulation levels reinforce the presence of coordinated physiological mechanisms underlying nutrient export. These findings support the potential to select genotypes with lower nutrient export while maintaining high productivity, thereby contributing to the development of more sustainable cultivation practices and reducing the reliance on external fertilizer inputs.

While this study focused on total nutrient export via whole fruits, it is noteworthy that coffee husks are often recycled locally as organic fertilizer. Future research could explore genotype-specific nutrient allocation patterns between husks and beans to optimize residue management and reduce external fertilization demands.

Utilizing nutrient accumulation information in genotypes can benefit both the productive and technological sectors. For the productive sector, the fruits represent the product of coffee cultivation and are harvested from the plants. Utilizing genotypes that have lower nutrient accumulation in the fruits for each ton of produced grains can benefit the production system by reducing the export of nutrients removed from the field. For the technological sector, nutritional information can be applied in research, such as in selecting parents for breeding programs aimed at developing coffee cultivars with greater nutritional efficiency. Thus, studying nutritional information and leveraging the existing variability in the species can promote the increase and maintenance of sustainability in coffee production.

## 5. Conclusions

This study revealed significant genetic variability among *C. canephora* genotypes with respect to nutrient accumulation in the fruits, highlighting the diversity present within the species. The results demonstrated that nitrogen (N) and potassium (K) are the most demanded nutrients by the fruits, underscoring the importance of efficient fertilizer management to ensure sustainable production. The genotypes Clementino and LB1 represented the extremes of highest and lowest nutrient export, respectively, providing valuable information for nutritional management and breeding programs. Multivariate analysis identified distinct groups of genotypes, with particular emphasis on Verdim R, Clementino, and Pirata, which formed isolated clusters due to their unique nutritional profiles. The high heritability of the evaluated traits, combined with the elevated genetic coefficient of variation, confirms that genetic control predominates in nutrient accumulation, suggesting that these traits can be effectively utilized in breeding programs. Ultimately, the application of the information generated by this study can contribute significantly to innovation in the coffee sector, both in production and research, thereby enhancing the competitiveness and sustainability of coffee cultivation.

## Figures and Tables

**Table 1:** Chemical and granulometric characterization of the soil in the experimental area, Nova Venécia, ES, Brazil.

Chemical Attributes	Depth (cm)					
	0–10	10–20	20–30	30–40	40–50	50–60
K (mg dm^−3^)	110.0	95.0	74.0	57.0	52.0	46.0
S (mg dm^−3^)	15.0	11.0	29.0	15.0	15.0	17.0
Ca (cmol dm^−3^)	3.8	3.4	1.9	1.0	0.7	0.6
Mg (cmol dm^3^)	1.0	0.9	0.4	0.3	0.1	0.1
Al (cmol dm^−3^)	0.0	0.0	0.3	0.7	0.8	0.8
H + Al	1.6	1.8	2.4	2.9	3.1	3.1
pH-H_2_O	6.6	6.5	5.3	4.8	4.8	4.8
Organic matter (dag dm^−3^)	2.1	1.7	1.1	0.8	0.7	0.5
Fe (mg dm^−3^)	140.0	138.0	126.0	94.0	88.0	87.0
Zn (mg dm^−3^)	10.2	4.5	2.9	1.1	0.6	0.5
Cu (mg dm^−3^)	3.4	4.3	3.0	1.9	1.2	1.0
Mn (mg dm^−3^)	207.0	174.0	104.0	46.0	44.0	40.0
B (mg dm^−3^)	0.8	0.8	0.6	0.6	0.6	0.6
Na (mg dm^−3^)	11.0	37.0	8.0	6.0	5.0	4.0
Granulometry (g kg^−1^)						
Sand	434.0	352.0	188.0	368.0	366.0	376.0
Silt	86.0	168.0	212.0	32.0	74.0	124.0.
Clay	480.0	480.0	600.0	600.0	560.0	500.0

pH: in H_2_O (1:2.5); P, K, Zn, Mn, Cu, and Fe (phosphorus, potassium, zinc, manganese, copper, and iron) extracted using Mehlich-1; S (sulfur): extracted using calcium monophosphate in acetic acid; Ca and Mg (calcium and magnesium): extracted using 1 mol/L KCl; Al + H (aluminum and hydrogen): determined by titration; organic matter: Embrapa method.

**Table 2:** Identification of the 42 *Coffea canephora* genotypes. Nova Venécia, ES, Brazil.

Identification	Name	Identification	Name	Identification	Name
1	Verdim R	15	Bamburral	29	Tardio C
2	B01	16	Pirata	30	A1
3	Bicudo	17	Peneirão	31	Cheique
4	Alecrim	18	Z39	32	P2
5	700	19	Z35	33	Emcapa 02
6	CH1	20	Z40	34	Emcapa 153
7	Imbigudinho	21	Z29	35	P1
8	AD1	22	Z38	36	LB1
9	Graudão HP	23	Z18	37	122
10	Valcir P	24	Z37	38	Verdim D
11	Beira Rio 8	25	Z21	39	Emcapa 143
12	Tardio V	26	Z36	40	Ouro negro 1
13	AP	27	Ouro Negro	41	Ouro negro 2
14	L80	28	18	42	Clementino

The treatments comprised 42 clonal genotypes of *C. canephora* var. Conilon (vegetatively propagated). Genotype 33 belongs to the Emcapa 8111 cultivar, while genotypes 34 and 39 belong to the Emcapa 8131 cultivar [27]. Genotypes 1, 11, 15, 16, and 30 belong to the Tributun cultivar; genotypes 30 and 35 to the Andina cultivar; and genotypes 9, 12, 24, 29, and 33 to the Salutar cultivar [28].

**Table 3:** Average nutrient accumulation in the fruits of 42 *Coffea canephora* genotypes for the production of one ton of processed coffee at 12% moisture in the 2017–2018 biennium.

Genotypes	N	P	K	Ca	Mg	S	Fe	Zn	Cu	Mn	B
	—————— kg·ton^−1^ ——————						————— g·ton^−1^ —————				
Verdim R	36.1 c	1.8 e	29.1 b	3.8 c	2.5 f	1.9 f	112.8 a	10.5 a	21.2 b	84.0 i	49.4 b
B01	39.0 a	1.5 j	30.7 a	4.1 c	2.9 c	1.9 f	92.2 c	9.4 c	16.4 g	166.8 c	53.4 a
Bicudo	34.8 d	1.7 f	31.6 a	4.0 c	2.4 f	1.9 f	75.3 g	10.0 b	14.8 h	92.2 i	49.2 b
Alecrim	34.9 d	1.7 f	25.6 d	4.7 b	2.6 e	2.1 c	74.4 g	8.8 d	21.5 b	134.8 f	45.9 c
700	33.2 e	1.5 i	24.9 d	3.7 d	2.3 g	1.8 h	79.0 f	8.1 f	19.2 c	171.6 c	36.4 f
CH1	34.4 d	1.6 h	25.1 d	3.6 d	2.9 c	1.9 e	91.2 c	8.3 e	19.2 c	163.4 d	51.7 a
Imbigudinho	34.0 e	1.7 f	26.4 c	3.4 e	2.7 d	1.7 i	74.1 g	8.1 f	19.1 c	133.4 f	41.1 d
AD1	31.3 f	1.8 e	26.0 c	2.8 f	2.1 i	1.6 i	81.9 e	8.0 f	15.8 g	88.8 i	39.5 e
Graudão HP	32.3 f	1.5 i	30.9 a	2.9 f	2.2 h	1.8 h	70.0 i	7.8 f	15.0 h	224.1 a	40.6 e
Valcir P	37.3 b	1.6 g	27.7 c	4.0 c	2.4 g	2.1 c	76.0 g	9.4 c	15.9 g	94.8 i	50.6 b
Beira Rio 8	37.1 b	1.7 f	28.8 b	4.9 a	2.4 f	2.0 e	84.5 d	9.2 d	16.2 g	142.4 e	51.8 a
Tardio V	31.4 f	1.6 g	26.8 c	4.1 b	2.2 h	1.7 i	65.5 j	8.9 d	13.7 i	114.4 g	40.8 e
AP	31.8 f	1.5 j	25.1 d	3.4 e	2.2 h	1.8 g	57.0 l	8.4 e	17.8 e	64.6 j	39.9 e
L80	36.0 c	1.6 h	28.0 b	4.6 b	2.4 g	1.8 g	69.8 i	9.8 b	20.6 b	89.3 i	41.9 d
Bamburral	34.7 d	1.5 j	25.8 d	4.3 b	2.6 e	2.0 e	72.7 h	9.4 c	16.8 f	216.4 a	45.3 c
Pirata	31.9 f	1.8 d	26.8 c	5.1 a	3.4 a	2.0 e	82.6 e	9.0 d	15.8 g	113.1 g	50.4 b
Peneirão	34.8 d	1.6 g	26.8 c	4.3 b	2.2 h	1.9 e	96.5 b	9.1 d	17.3 f	115.0 g	38.3 f
Z39	33.3 e	1.8 d	26.6 c	3.6 d	2.4 f	1.7 h	75.1 g	7.8 f	20.8 b	170.1 c	44.6 c
Z35	35.4 d	1.8 e	26.7 c	4.7 b	2.2 h	2.1 d	86.5 d	8.5 e	15.1 h	71.4 j	41.9 d
Z40	37.4 b	1.6 i	27.2 c	4.6 b	2.2 h	2.2 c	72.8 h	7.9 f	14.8 h	114.9 g	35.0 g
Z29	37.4 b	1.8 d	25.4 d	4.4 b	2.6 e	1.9 e	69.5 i	7.6 g	19.4 c	138.0 e	44.4 c
Z38	35.1 d	1.5 j	26.9 c	4.6 b	2.6 e	2.2 c	67.1 j	7.5 g	18.6 d	117.6 g	36.3 f
Z18	32.2 f	1.6 h	27.6 c	4.3 b	2.2 h	2.0 d	66.9 j	7.8 f	19.1 c	126.8 f	40.7 e
Z37	36.1 c	2.1 a	32.1 a	4.9 a	2.4 f	2.2 c	83.6 e	7.9 f	21.3 b	120.2 g	48.1 b
Z21	32.7 e	1.6 g	25.3 d	3.8 d	2.3 h	1.7 h	72.9 h	7.5 g	17.9 e	75.9 j	39.4 e
Z36	31.5 f	1.5 j	26.9 c	4.5 b	2.1 i	1.8 g	73.4 h	7.9 f	18.7 d	84.9 i	42.8 d
Ouro Negro	33.4 e	1.6 i	30.5 a	4.0 c	2.0 i	1.9 e	61.4 k	7.1 g	16.9 f	226.4 a	32.3 g
18	39.0 a	1.8 d	29.5 b	5.0 a	2.4 g	2.1 d	55.9 l	8.2 f	16.4 g	103.9 h	52.0 a
Tardio C	34.9 d	1.5 i	27.8 b	4.9 a	2.5 f	2.4 a	58.5 l	7.8 f	14.2 h	149.1 e	42.3 d
A1	35.8 c	1.7 f	29.0 b	4.5 b	2.2 h	2.0 d	66.8 j	6.1 i	12.6 j	94.0 i	51.0 a
Cheique	39.3 a	1.9 c	29.1 b	4.4 b	2.4 g	2.2 b	78.9 f	7.3 g	19.5 c	89.2 i	39.6 e
P2	34.7 d	1.5 i	26.4 c	4.4 b	2.0 i	1.8 g	67.2 j	6.3 i	18.1 e	143.2 e	42.1 d
Emcapa 02	33.2 e	1.5 j	25.6 d	3.7 d	2.0 i	1.7 i	51.1 m	6.0 i	18.7 d	119.4 g	39.1 e
Emcapa 153	32.6 e	1.7 f	27.4 c	5.1 a	2.4 g	2.0 e	63.8 k	6.9 h	24.2 a	157.1 d	45.7 c
P1	35.2 d	1.6 h	25.2 d	4.3 b	2.1 i	1.9 e	67.7 j	7.4 g	20.2 c	188.2 b	38.1 f
LB1	29.5 g	1.5 i	22.8 e	4.3 b	2.2 h	1.6 i	50.5 m	5.9 i	17.9 e	171.6 c	35.2 g
122	35.6 c	1.7 f	28.2 b	5.1 a	2.4 f	2.2 b	59.1 l	7.2 g	18.7 d	108.9 h	43.2 d
Verdim D	34.8 d	1.6 i	25.4 d	4.6 b	2.2 h	2.0 e	62.0 k	6.2 i	19.7 c	140.8 e	42.4 d
143	32.1 f	1.6 g	25.2 d	4.4 b	2.5 e	1.9 f	71.0 h	7.5 g	18.2 e	146.2 e	39.4 e
Ouro negro 1	35.2 d	1.8 e	23.2 e	3.9 c	2.5 e	2.0 e	65.9 j	6.1 i	19.1 c	108.1 h	49.3 b
Ouro negro 3	33.9 e	1.7 e	25.0 d	4.5 b	2.1 h	1.7 h	83.0 e	6.8 h	21.2 b	131.5 f	45.1 c
Clementino	39.6 a	2.0 b	28.3 b	4.3 b	3.1 b	2.4 a	110.2 a	8.1 f	24.9 a	146.0 e	50.0 b
	Summary of variance analysis										
Genotypes	47.6 **	84.6 **	18.4 **	20.9 **	57.9 **	35.2 **	175.4 **	77.9 **	87.8 **	144.9 **	27.4 **
CVe (%)	1.9	1.9	3.7	5.7	3.2	3.4	2.8	3.2	3.1	5.1	4.8
Média	34.6	1.7	27.1	4.3	2.4	1.9	73.7	7.9	18.2	129.8	43.5

Means followed by the same letter in the column do not differ according to the Scott–Knott test at a 5% probability level. ** Significant at a 1% probability level according to the F test., CVe: coefficient of experimental variation.

**Table 4:** Estimates of the coefficient of experimental variation (CVe), genetic variation coefficient (CVg), and genotypic determination coefficient (H^2^) for nutrient accumulation in the fruits of 42 *Coffea canephora* genotypes, considering the production of beans during the 2017–2018 biennium.

Nutrients	CVe (%)	CVg (%)	H^2^ (%)
N	1.98	6.75	97.90
P	1.86	8.53	98.83
K	3.67	7.64	94.55
Ca	5.69	12.70	95.22
Mg	3.17	11.95	98.28
S	3.44	10.04	97.15
Cu	3.12	14.54	98.86
Fe	2.79	18.40	99.43
Mn	5.11	30.66	99.31
Zn	3.24	14.21	98.72
B	4.76	12.23	96.35

CVe: coefficient of experimental variation, CVg: coefficient of genetic variation, H^2^: genotypic determination coefficient.

**Table 5:** Relative contribution of nutrient accumulation in the fruits to genetic diversity in 42 *Coffea canephora* genotypes, according to the Singh method (1981).

Nutrients	S.j. ^1^	Contribution %	Accumulated Value
Mn	2,747,064.9	87.2	87.2
Fe	318,796.0	10.1	97.3
B	50,556.6	1.6	98.9
Cu	12,128.1	0.4	99.3
N	9627.1	0.3	99.6
K	7824.8	0.3	99.9
Zn	2215.8	0.1	99.9
Ca	528.1	0.1	99.9
Mg	14.2	0.0	100.0
S	67.1	0.0	100.0
P	34.6	0.0	100.0

^1^ S.j ([31]).

## Data Availability

The original contributions presented in this study are included in the article. Further inquiries can be directed to the corresponding authors.
